# Endotyping Eosinophilic Inflammation in COPD with ELAVL1, ZfP36 and HNRNPD mRNA Genes

**DOI:** 10.3390/jcm13030854

**Published:** 2024-02-01

**Authors:** Ilektra Voulgareli, Maria Semitekolou, Ioannis Morianos, Myrto Blizou, Maria Sfika, Georgios Hillas, Petros Bakakos, Stelios Loukides

**Affiliations:** 1 2nd Respiratory Medicine Department, “Attikon” University Hospital, National and Kapodistrian University of Athens Medical School, 11527 Athens, Greece; ilektravoul@gmail.com (I.V.); myrto_bl@hotmail.com (M.B.); maria.sfka@gmail.com (M.S.); 2 School of Medicine, Institute of Molecular Biology and Biotechnology, University of Crete, Foundation for Research and Technology—Hellas Voutes, 71110 Heraklion, Crete, Greece; msemi@bioacademy.gr (M.S.); jmor@bioacademy.gr (I.M.); 3 5th Respiratory Medicine Department, “Sotiria” Chest Hospital, 11527 Athens, Greece; ghillas70@yahoo.gr; 4 1st Respiratory Medicine Department, “Sotiria” Chest Hospital, National and Kapodistrian University of Athens Medical School, 11527 Athens, Greece; petros44@hotmail.com

**Keywords:** HuR, TTP, AUF-1, IL-9, IL-33, COPD, eosinophilic, inflammation

## Abstract

**Background**: Chronic obstructive pulmonary disease (COPD) is a common disease characterized by progressive airflow obstruction, influenced by genetic and environmental factors. Eosinophils have been implicated in COPD pathogenesis, prompting the categorization into eosinophilic and non-eosinophilic endotypes. This study explores the association between eosinophilic inflammation and mRNA expression of ELAVL1, ZfP36, and HNRNPD genes, which encode HuR, TTP and AUF-1 proteins, respectively. Additionally, it investigates the expression of IL-9 and IL-33 in COPD patients with distinct eosinophilic profiles. Understanding these molecular associations could offer insights into COPD heterogeneity and provide potential therapeutic targets. **Methods**: We investigated 50 COPD patients, of whom 21 had eosinophilic inflammation and 29 had non-eosinophilic inflammation. Epidemiological data, comorbidities, and pulmonary function tests were recorded. Peripheral blood mononuclear cells were isolated for mRNA analysis of ELAVL1, ZfP36, and HNRNPD genes and serum cytokines (IL-9, IL-33) were measured using ELISA kits. **Results**: The study comprised 50 participants, with 66% being male and a mean age of 68 years (SD: 8.9 years). Analysis of ELAVL1 gene expression revealed a 0.45-fold increase in non-eosinophilic and a 3.93-fold increase in eosinophilic inflammation (*p* = 0.11). For the ZfP36 gene, expression was 6.19-fold higher in non-eosinophilic and 119.4-fold higher in eosinophilic groups (*p* = 0.07). Similarly, HNRNPD gene expression was 0.23-fold higher in non-eosinophilic and 0.72-fold higher in eosinophilic inflammation (*p* = 0.06). Furthermore, serum levels of IL-9 showed no statistically significant difference between the eosinophilic and non-eosinophilic group (58.03 pg/mL vs. 52.55 pg/mL, *p* = 0.98). Additionally, there was no significant difference in IL-33 serum levels between COPD patients with eosinophilic inflammation and those with non-eosinophilic inflammation (39.61 pg/mL vs. 37.94 pg/mL, *p* = 0.72). **Conclusions**: The data suggest a notable trend, lacking statistical significance, towards higher mRNA expression for the ZfP36 and HNRNPD genes for COPD patients with eosinophilic inflammation compared to those with non-eosinophilic inflammation.

## 1. Introduction

Chronic obstructive pulmonary disease (COPD) is a prevalent and manageable condition that affects both the airways and lung tissue [1]. It is primarily characterized by progressive airflow obstruction, mainly caused by factors such as cigarette smoke and environmental pollutants [1]. The global burden of COPD is on the rise, impacting more than 200 million individuals [1].

Its development is influenced by a complex interplay of genetic and environmental factors. In COPD, prolonged lung inflammation triggered by environmental factors leads to the infiltration of immune cells, including neutrophils, monocytes, TH1, and TH17 cells, into the bronchial airways [2]. Recent research has highlighted increased levels of eosinophils in sputum of both stable COPD patients and those experiencing exacerbations [2]. This suggests a potential role for eosinophils in the disease’s pathogenesis, leading to the categorization of COPD into eosinophilic COPD and non-eosinophilic COPD in various studies [2].

Researchers have been investigating the differential expression of various cytokines in COPD patients with eosinophilic inflammation and non-eosinophilic inflammation [3]. Among the cytokines studied, IL-33 and IL-9 have garnered particular attention [3,4].

IL-33, a relatively recent discovery in the IL-1 family, serves as an alarmin cell cytokine that promotes inflammatory responses [3]. This cytokine promotes the Th2 immune response and induces the production of pro-inflammatory cytokines and chemokines, including IL-4, IL-5, IL-13, TNF-α, GM-CSF, CCL2, and prostaglandin D23. IL-33 also triggers eosinophil degranulation and the production of reactive oxygen species (ROS) [3].

As for IL-9, it is primarily produced by Th9 cells, which derive from CD4+ T lymphocytes upon stimulation by TGF-β and IL-4 [4]. It has been found to enhance the mRNA expression of the IgE receptor on histiocytes’ cell surface and stimulate mast cells to produce cytokines that promote Th2 inflammation [4]. Elevated levels of IL-9 have been reported in peripheral blood, bronchoalveolar lavage, and lung tissue of mice exposed to cigarette smoke, as well as in the sputum of COPD patients, suggesting its potential involvement in COPD’s pathogenesis [4].

RNA-binding proteins (RBPs) constitute an important group of regulatory transcription factors involved in various cellular processes, particularly primary immunity [5]. Three RBPs—human antigen R (HuR), tristetraprolin (TTP), and AU-rich element binding factor 1 (AUF-1)—have been investigated in the context of COPD.

HuR is known to regulate the transcription of numerous cytokines, including those involved in Th2/Th17 differentiation, by stabilizing their mRNA [6]. Increased levels of HuR result in an upregulation of Th2 cytokines, including IL-4 and IL-13, promoting the Th2 immune response [5].

TTP has been shown to destabilize mRNA associated with processes like airway smooth muscle hyperplasia, inflammation, bronchial wall thickening, and angiogenesis [6]. This leads to a mononuclear inflammatory response, mucus plugging, goblet cell metaplasia, smooth muscle hyperplasia, and ultimately airflow limitation [6].

AUF-1, which exists in four isoforms, has been studied in COPD patients [7]. Research has shown a significant reduction in AUF-1 levels in the bronchial epithelium of COPD patients compared to controls, potentially implicating reduced AUF-1 in the pathogenesis of COPD [7].

The aim of our study was to investigate the association between eosinophilic inflammation and the levels of mRNA expression for the ELAVL1, ZfP36 and HNRNPD genes, which encode the HuR, TTP and AUF-1 proteins, respectively. Moreover, we tried to investigate whether cytokines IL-9 and IL-33 are expressed differently in patients with COPD characterized by eosinophilic vs. non eosinophilic inflammation.

## 2. Materials and Methods

### 2.1. Patients

Patients were recruited from three respiratory outpatient departments: the 1st University Respiratory Department in Sotiria Hospital, 2nd University Respiratory Department in Attikon Hospital and 5th Respiratory Department in Sotiria Hospital. The recruitment of patients started in May 2022 and ended in June 2023. A total of 50 patients were included in the study. The diagnosis of COPD was based on the GOLD 2023 report [1]. All participants in the study were over the age of 40, had a smoking history of more than 10 pack-years, demonstrated a post-bronchodilator forced expiratory volume in 1 s (FEV1)/forced vital capacity (FVC) ratio less than 0.7, and showed no significant abnormalities on chest radiography. Subjects with other respiratory diseases or significant comorbidities were excluded from the study, as were subjects with COPD exacerbation or respiratory tract infection in the 8 weeks preceding the study. Subjects were divided in those with eosinophilic COPD (those who had three consecutive measurements with blood eosinophils > 4% and/or >300/μL in absolute count) and those with non-eosinophilic COPD that did not fulfill the above criterion. Demographics, comorbidities and COPD medication were recorded by treating physicians. The study was approved by the scientific committee of Sotiria hospital (2321/30-1-20). All participants provided written informed consent.

### 2.2. Isolation of Peripheral Blood Mononuclear Cells (PBMCs)

The mRNA of ELAVL1, ZfP36 and HNRNPD genes was analyzed in peripheral blood mononuclear cell samples (PBMCs). PBMCs were obtained using Histopaque (Sigma-Aldrich) density-gradient centrifugation. PBMCs were stored at −80 °C for further experimentation.

### 2.3. Quantitative Real-Time PCR

Total RNA was isolated by using the QiagenRNeasy Protect Cell Mini Kit and reverse transcribed with PrimeScript™ RT reagent Kit (Perfect Real Time), according to the manufacturer’s recommendations. Gene expression was analyzed using SYBR Green Master mix and selected primers (Table 1). The relative expression of genes over the expression of Gapdh and Polr2a was calculated by using the 2^−ΔΔCt^ analysis method [8].

### 2.4. Cytokine Analysis

Cytokines were measured in the sera of COPD individuals using commercially available ELISA kits for human IL-9 and IL-33 (R&D Systems, Minneapolis, MN, USA).

### 2.5. Statistical Analysis

Categorical variables are presented as n (%), whereas numerical variables are presented as mean ± standard deviation (SD) or median (interquartile ranges) for normally distributed and skewed data, respectively. Normality of distributions was checked with Kolmogorov–Smirnov test. Comparisons between groups were performed using chi-square tests for categorical data, as well as unpaired *t*-tests or Mann–Whitney U-tests for normally distributed or skewed numerical data. Correlations were assessed using Spearman’s and Pearson’s correlation coefficients for skewed and normally distributed variables, respectively. Graph Pad Prism 9 (Graph Pad Software Inc.) was used for all statistical analyses. We considered any difference with a *p* value of 0.05 or less to be statistically significant.

## 3. Results

A total of 50 participants were enrolled and included in this study with a mean age of 68 years (SD:8.9); 21 patients were characterized by eosinophilic inflammation and 29 patients were characterized by non-eosinophilic inflammation. The demographic and clinical characteristics for the study population are shown in Table 2.

**Figure 1:**
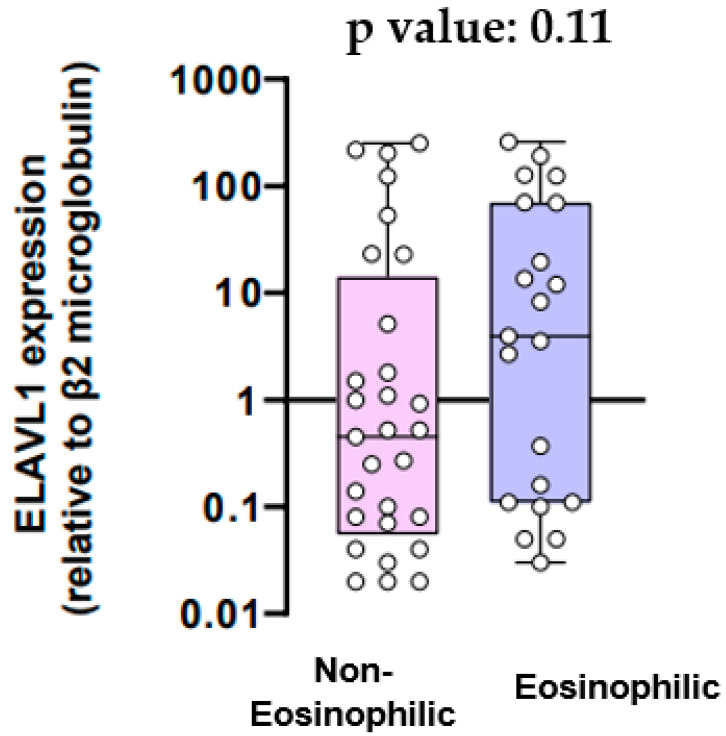
ELAVL1 expression (relative to β2 microglobulin).

mRNA expression for the ELAVL1 gene in the eosinophilic and non-eosinophilic populations is provided in Figure 1. The mean mRNA expression for the ELAVL1 gene was 0.45-fold higher relative to β2 microglobulin in patients with non-eosinophilic inflammation and 3.93-fold higher in patients with eosinophilic inflammation (*p* value = 0.11).

**Figure 2:**
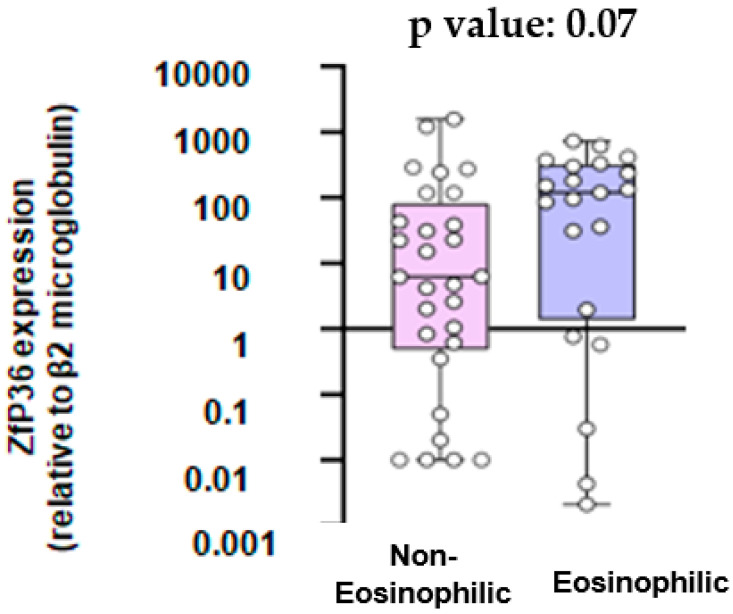
ZfP36 expression (relative to β2 microglobulin).

As shown in Figure 2, the mean mRNA expression for the ZPf36 gene was 6.19-fold higher relative to β2 microglobulin in the non-eosinophilic group and 119.4-fold higher in the eosinophilic group (*p* value = 0.07).

**Figure 3:**
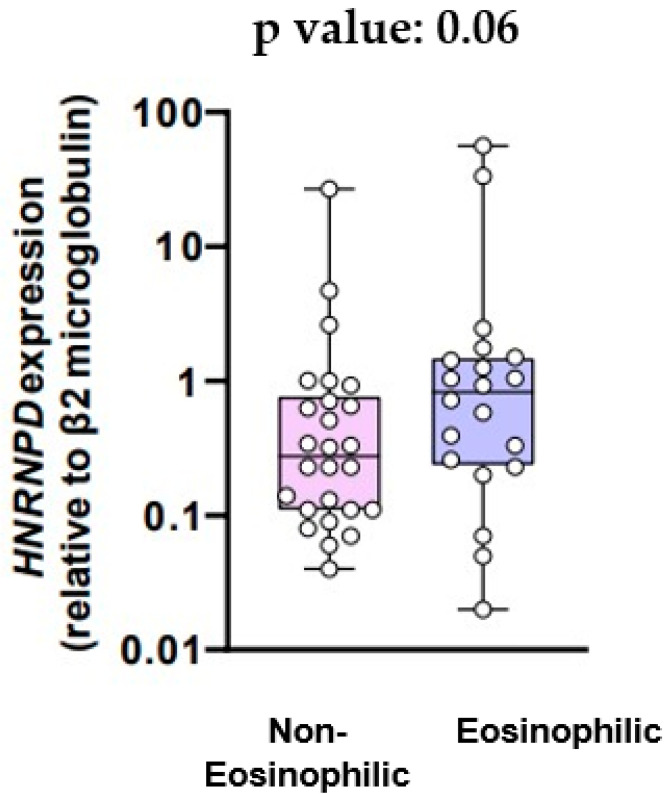
HNRNPD expression (relative to β2 microglobulin).

The mean mRNA expression for the HNRNPD gene in the two groups is provided in Figure 3. The mean mRNA expression was 0.23-fold higher relative to β2 microglobulin in patients with non-eosinophilic inflammation and 0.72-fold higher in patients with eosinophilic inflammation (*p* value = 0.06).

**Figure 4:**
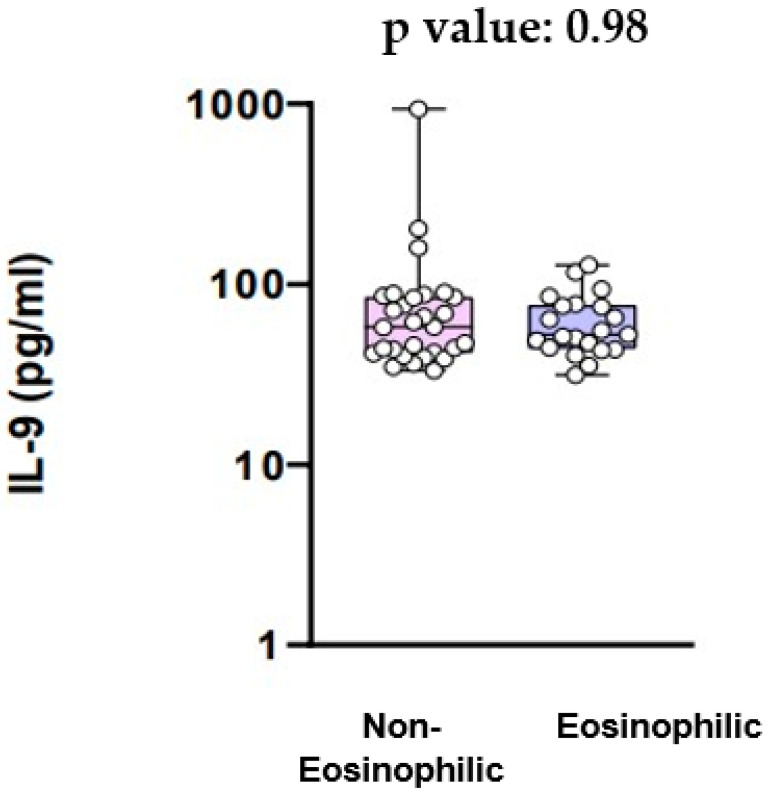
IL-9 serum levels.

The levels of IL-9 are shown in Figure 4. IL-9 serum levels did not have a statistically significant difference between the eosinophilic and the non-eosinophilic COPD groups (58.03 pg/mL vs. 52.55 pg/mL, *p* value = 0.98) (Figure 4).

**Figure 5:**
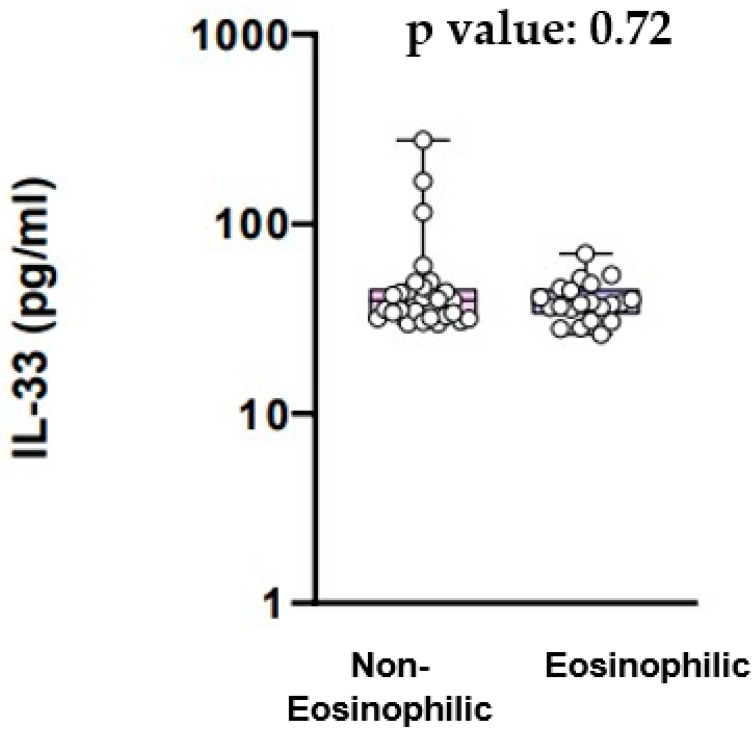
IL-33 serum levels.

The levels of IL-33 are provided in Figure 5. IL-33 serum levels did not differ significantly between the COPD patients with eosinophilic inflammation and the COPD patients with non-eosinophilic inflammation (39.61 pg/mL vs. 37.94 pg/mL, *p* value = 0.72) (Figure 5).

## 4. Discussion

The inflammatory pattern in COPD can vary, often characterized by a predominance of neutrophils, cytotoxic CD8+ T cells, and alveolar macrophages [2]. Eosinophils may play a significant role in airway inflammation for certain COPD patients [2]. While eosinophilic inflammation is typically associated with asthma and used to differentiate it from COPD, studies suggest that around a third of COPD patients exhibit sputum eosinophilia, with the prevalence influenced by the eosinophil threshold and the specific patient population studied [2]. Additionally, during exacerbations in some COPD patients, there is an increase in eosinophil counts in sputum [2]. While there is no universally established threshold to define eosinophilic inflammation in the context of COPD, increased eosinophil counts have been linked to decreased lung function and an increased risk of exacerbations in COPD patients [2]. Furthermore, a decrease in eosinophilic inflammation is correlated with a reduction in the frequency of exacerbations [2]. Elevated eosinophil counts in the bloodstream could serve as an acceptable surrogate for airway eosinophilia [2]. Notably, eosinophilia in sputum and blood in COPD may indicate a potential response to inhaled corticosteroids (ICSs) for preventing exacerbations and to systemic corticosteroids (CSs) for treating exacerbations [2]. In COPD, emerging therapies targeting eosinophil chemotactic and survival factors, including monoclonal antibodies directed at the IL5 ligand, IL5 receptor, IL4 receptor, and IL13 ligand, have been investigated [2]. Some of these therapies, originally studied in asthma, are now being explored in COPD and may demonstrate effectiveness as treatments [2].

Our study’s objective was to examine the relationship between eosinophilic inflammation in COPD and the mRNA expression levels of genes ELAVL1, ZfP36, and HNRNPD, responsible for encoding the HuR, TTP, and AUF-1 proteins, respectively. First of all, our results showed that the mRNA expression for the ELAVL1 gene did not differ significantly between COPD patients with eosinophilic inflammation and COPD patients with non-eosinophilic inflammation. On the other hand, there was a trend towards higher mRNA expression of the Zfp36 gene and the HNRNPD gene in patients with eosinophilic inflammation compared to those with non-eosinophilic inflammation, although this was not statistically significant. Additionally, we aimed to explore potential variations in the expression of cytokines IL-9 and IL-33 between COPD patients exhibiting eosinophilic and non-eosinophilic inflammation. We found that serum IL-9 levels did not exhibit a statistically significant difference between the eosinophilic and the non-eosinophilic group. Likewise, there was no significant difference in IL-33 serum levels between COPD patients with eosinophilic inflammation and those with non-eosinophilic inflammation.

RNA-binding proteins (RBP) play a crucial role in regulating various aspects of RNA metabolism, including biogenesis, cellular localization and transport, stability and translation [9]. The advent of high-throughput screening and quantitative proteomics has led to the identification of approximately 500 potential RNA-binding proteins (RBPs) [9]. Recognizing their significant importance, substantial efforts have been devoted to expanding our understanding of how RNA–protein interactions influence RNA function and cell fate. It is crucial to emphasize that mRNA, far from being a simple rod, adopts a complex three-dimensional structure [9]. Consequently, RBPs can engage with mRNA through structural, sequence, or structural and sequence elements [9]. Some RBPs are likely to become viable targets for therapeutics in the treatment of human diseases [9]. Upon transcription from genomic DNA, newly synthesized pre-mRNAs are promptly enveloped by nuclear RBPs, safeguarding them from degradation by nucleases, guiding splicing, and preparing them for cytoplasmic transport [9]. As mature mRNAs move to the cytoplasm, the repertoire of bound proteins is often replaced by a new set of RBPs that dictate intracellular location, define degradation rates, and regulate translatability [9]. In response to external and internal stimuli, both free and bound RBPs undergo post-translational modifications (PTMs) such as phosphorylation, ubiquitination, acetylation, and methylation [9]. These PTMs may induce conformational changes and modify the association between RBP and target mRNA [9]. Depending on the stimulus and cell type, modified RBPs may associate with or dissociate from mRNA, influencing transcript stability and translation, thereby impacting protein production [9]. RBPs bind to RNA through various domains, including the RNA-recognition motif (RRM), zinc finger motifs, K-homology domains (KH), RGG boxes, and DEAD/DEAH boxes [9]. Often, multiple binding domains are present, enabling simultaneous interactions with multiple mRNAs, multiple sites within one mRNA target, or specific mRNA sequences and organelles such as ribosomes or stress granules [9]. Three of the most important RNA-binding proteins (RBPs) described are HuR, TTP, and AUF-1.

HuR, encoded from the ELAVL1 gene, is a member of the embryonic lethal abnormal vision (ELAV) family of RNA-binding proteins (RBPs) [10]. It engages with mRNAs, influencing their stability and/or translation, and is involved in processes such as pre-mRNA splicing and the nucleocytoplasmic translocation of target mRNAs [11,12]. The encoded proteins from these targeted mRNAs play roles in cellular functions like proliferation, differentiation, apoptosis, and inflammation [10,11]. It is known that pulmonary macrophages and fibroblasts in COPD patients exhibit a relatively elevated level of HuR expression in the cytoplasm [13]. Also, it has been discovered that there is increased expression of HuR in the airway epithelium among COPD patients [14]. Specifically, it has been found that in non-COPD smokers, the expression levels of HuR are higher compared to nonsmokers [14]. Furthermore, smokers with COPD seem to exhibit notably elevated levels of HuR compared to both non-smokers and smokers without COPD [14]. This observation suggests a significant involvement of HuR in the pathogenesis of COPD, particularly linked to exposure to cigarette smoke [14]. Moreover, research confirms altered expression of HuR in human bronchial epithelial cells after cigarette smoke extract exposure [14] and also suggests that beyond the increased expression of HuR, cigarette smoke exposure can facilitate the translocation of HuR from the nucleus to the cytoplasm [14]. HuR, as a prominent RNA-binding protein, is primarily localized in the nucleus during cellular quiescence, and upon activation, HuR swiftly translocates from the nucleus to the cytoplasm, where it exerts its RNA-binding activities and the increased cytoplasmatic HuR levels may imply that cigarette smoke exposure could enhance the activities of HuR [14]. Also, findings from mouse experiments indicated that selectively eliminating HuR in T cells hinders Th2 differentiation [15]. Conversely, Baker et al. [16] found that there was a notable decrease in both mRNA and protein levels of HuR in peripheral lung samples obtained from patients with COPD. Furthermore, the association of HuR with eosinophils is evident, as it seems that eosinophils express the RBP HuR [17]. It has also been found that HuR expression is elevated in nasal polyp tissues, particularly in those characterized by eosinophilic features [18]. Herjan et al. [19] also indicated that the deletion of HuR in the airway epithelium attenuated pulmonary inflammation induced by house dust mist, primarily by decreasing the levels of the eosinophil-specific chemoattractant CCL. However, there was no alteration observed in Th2 cytokines and analysis of bronchoalveolar lavage (BAL) fluid and lung histology revealed a substantial reduction in eosinophil infiltration following the airway epithelial-specific deletion of HuR [19]. Considering HuR’s participation in COPD pathogenesis and its interaction with eosinophils, we hypothesized that there could be different mRNA expression patterns for the ELAVL1 gene in patients with eosinophilic COPD compared to those with non-eosinophilic COPD. However, our study did not establish a statistically significant difference.

TTP, encoded by the Zfp36 gene, plays a vital role as an anti-inflammatory protein, facilitating mRNA decay [20]. Post-transcriptional mRNA-stabilizing mechanisms regulate numerous clinically significant proteins, including cytokines, like GM-CSF, which is found to increase levels of eosinophils [20]. Notably, TTP is implicated in the development of COPD as, in COPD patients, an increase in phosphorylated p38 MAPK-positive cells in the alveolar region activates the inflammatory pathway via p38 MAPK, which, facilitated by MAPK kinase 2 (MK2), phosphorylates and subsequently inactivates TTP [21]. Moreover, many of the mRNAs targeted by TTP encode proteins associated with processes such as thickening of the basement membrane, heightened deposition of the interstitial matrix, augmentation of airway smooth muscle mass, increased airway fibrosis, and neovascularization in the subepithelial mucosa, which are involved in airway inflammation [20]. Considering the role of TTP in COPD pathogenesis and its interaction with various inflammatory cells, including eosinophils, we posited that there might be different mRNA expression patterns of the Zfp36 gene in patients with eosinophilic COPD compared to patients with non-eosinophilic COPD. While the observed trend did not achieve statistical significance, there was a discernible pattern suggesting that patients with COPD and eosinophilic inflammation tended to display elevated mRNA expression of the Zfp36 gene and consequently most likely higher levels of TTP.

AUF-1, encoded by the HeteronuclearRibonucleoprotein D (HNRNPD) gene, is a member of a widely expressed protein family primarily involved in facilitating the degradation of mRNA targets [22]. AUF1 undergoes post-transcriptional alternative splicing events, resulting in four distinct AUF1 mRNA isoforms (p37, p40, p42, and p45), all observable in human eosinophils [23]. Notably, the p37 isoform has demonstrated interaction with the exosome within eosinophils [24]. Furthermore, in silico analysis has documented alterations in AUF-1 expression in COPD [25]. This was observed in the context of a global downregulation trend across a curated list of 600 RNA-binding proteins (RBPs) in two transcriptomic databases focusing on the COPD bronchiolar epithelium [26]. The identified downregulated expression pattern of RBPs, including AUF-1, holds significance as it was found to be notably associated with various pathogenic pathways implicated in COPD [27]. Given the fact that AUF-1 plays a role in the pathogenesis of COPD and also interacts with eosinophils, we hypothesized that there might be differential mRNA expression of the HNRNPD gene between patients with eosinophilic COPD and those with non-eosinophilic COPD. Although it did not reach the threshold of statistical significance, there was indeed a noticeable trend indicating that COPD patients with eosinophilic inflammation exhibited higher mRNA expression of the HNRNPD gene and, by extension, higher levels of AUF-1. It is known that patients with COPD exhibit elevated levels of IL-9 compared to healthy individuals [28]. Moreover, it is a fact that the expression of biologically active IL-9 by human eosinophils implies their potential role in influencing the recruitment and activation of cells associated with the pathogenesis of various eosinophilic diseases [29]. IL-9, initially identified as a TH2-type cytokine with T-cell and mast-cell growth factor properties, has recently gained recognition for its role in asthma and allergies [29]. Emerging evidence supports its pleiotropic effects on cell types associated with allergic diseases, including TH2-type lymphocytes, mast cells, B cells, eosinophils, and airway epithelial cells [29]. Notably, IL-9′s biological functions encompass the promotion of mast cell proliferation and differentiation, as well as the enhancement of IgE production in B cells [29]. In recent studies using IL-9 transgenic mice, in vivo expression of IL-9 was found to lead to increased airway hyperresponsiveness, mimicking features observed in human asthma [29]. These models exhibited mucous hypersecretion, basement membrane thickening, heightened intraepithelial mast cell numbers, lung eosinophilia, elevated IgE levels, airway hyperreactivity, and increased sensitivity to antigen stimulation [29]. Furthermore, various studies have highlighted a robust correlation between the extent of inflammation, particularly eosinophilia, and airway hyperreactivity in individuals with asthma [29]. Finally, in asthma animal models, IL-9 regulates airway inflammation, mucus production, airway hyperresponsiveness, and airway fibrosis [30]. However, there is limited data on whether IL-9 levels differ between COPD patients with eosinophilic inflammation and those with non-eosinophilic inflammation. Our study did not demonstrate a statistically significant difference.

Generally, it is known that IL-33 appears to have significant implications in eosinophil-driven inflammation as it can stimulate eosinophils to generate superoxide anions and undergo degranulation and can enhance the survival of eosinophils [31]. IL-33 was identified through bioinformatic analysis of the human genome and is currently classified as a novel member of the IL-1 cytokine family, designated as IL-1F11/IL-33 [31]. Typically, IL-1 family members are broadly expressed in hematopoietic cells and play crucial roles in inflammatory responses and host defenses [31]. However, human IL-33 demonstrates a more restricted expression pattern, primarily found in epithelial cells from the bronchus and small airways, fibroblasts, and smooth muscle cells [31]. This suggests its involvement in the regulation of mucosal organ function [31]. Indeed, mice injected with IL-33 exhibit significant pathological changes in mucosal tissues, including eosinophilia in the lung, esophagus, and intestine, as well as elevated serum levels of IgA and IgE. Our findings regarding IL-33 are in contrast with the study by Tworek et al. [32], which showed that IL-33 levels were higher in COPD subjects with sputum eosinophilia than in those without. Likewise, Kim et al. [33] found that the serum levels of IL-33 in patients with stable COPD showed an association with the eosinophil count.

Our study had some limitations. First of all, it is possible that our findings would differ if we assessed the RBPS, IL-9 and IL-33 expression locally such as in sputum samples or in exhaled breath condensate (EBC). Additionally, the sample size was modest. Moreover, COPD exhibits heterogeneity, with its traditional classification into chronic bronchitis and emphysematous type, so it is possible that if we had provided deeper insights into the clinical phenotypes of COPD patients, we could have had different results. Hence, multicenter investigations with more participants are required. Finally, there is a lack of a control group of normal subjects.

## 5. Conclusions

Our data suggest that the mRNA expression of ZfP36 and HNRNPD genes demonstrates a noticeable trend toward higher expression among COPD patients with eosinophilic inflammation compared to those with non-eosinophilic inflammation, although lacking statistical significance. However, the mRNA expression for the ELAVL1 gene and the levels of IL-9 and IL-33 did not show any association with eosinophilic inflammation in COPD. Further investigation with an expanded sample size and a well-designed study is essential to confirm the above findings.

## Figures and Tables

**Table 1:** Primers that were used to analyze gene expression.

Primers	
**ELAVL1**:	
Forwardprimer	GGCGCAGAGATTCAGGTTCT
Reverseprimer	TGGTCACAAAGCCAAACCCT
**ZfP36**:	
Forwardprimer	ACTGCCATCTACGAGAGCCT
Reverseprimer	CACTAGGCTGGTGGAGCG
**HNRNPD**:	
Forwardprimer	TATCCAGGCGAGGTGGTCAT
Reverseprimer	GAAAATACTGCTTCACCACCTGTT
**beta-2 microglobulin:**	
Forwardprimer	TGAGTATGCCTGCCGTGTGA
Reverseprimer	TGATGCTGCTTACATGTCTCGAT

**Table 2:** Demographic and clinical characteristics of study participants.

Variables	All Patients n = 50	Non-Eosinophilic (Eosinophils < 4% and/or 300/μL) n = 29	Eosinophilic (Eosinophils 4% and/or 300/μL) n = 21	*p* Value
**Sex, Male**	33 (66%)	16 (55.2%)	17 (81%)	0.058
**Age, Years**	68 ± 8.9	68 ± 8.3	68 ± 9.9	0.989
**BMI, kg/m^2^**	27 (23.8–29.8)	24.5 (23.4–30.2)	28.1 (24.8–29.8)	0.371
**Smoker (N, %)**	32 (64%)	18 (66%)	13 (62%)	0.629
**FEV1, Liters**	1.5 ± 0.51	1.44 ± 0.53	1.58 ± 0.46	0.343
**FEV1, % of predicted**	60.7 ± 18.9	61.1 ± 20.7	60.1 ± 16.7	0.862
**FEV1/FVC, %**	57.8 ± 11.9	57.9 ± 12.7	57.6 ± 11.1	0.922
**Comorbidities**				
Coronary Artery Disease	7 (14%)	6 (20.7%)	1 (4.8%)	0.109
Hypertension	25 (50%)	13 (44.8%)	12 (57.1%)	0.39
Diabetes	10 (20%)	4 (13.8%)	6 (28.6%)	0.197
Atrial Fibrillation	5 (10%)	2 (6.9%)	3 (14.3%)	0.39
Ischemic Stroke	5 (10%)	2 (6.9%)	3 (14.3%)	0.39
Heart failure	2 (4%)	1 (3.4%)	1 (4.7%)	0.815
Cancer	11 (22%)	6 (20.7%)	5 (23.8%)	0.793
Connective Tissue Disease (CTD)	4 (8%)	3 (10.3%)	1 (4.7)	0.473
**Therapy**				
LAMA	6 (12%)	4 (13.8%)	2 (9.5%)	0.647
LABA/LAMA	15 (30%)	10 (34.5%)	5 (23.8%)	0.416
LABA/ICS	4 (8%)	1 (3.4%)	3 (14.3%)	0.163
LABA/LAMA/ICS	21 (42%)	12 (41.4%)	9 (42.9%)	0.917
No treatment	4 (8%)	2 (6.9%)	2 (9.5%)	0.735

Data are presented as mean ± standard deviation (SD) or median (interquartile ranges). *p* values indicate differences between the 2 groups. Abbreviations: BMI: body mass index; FEV1: forced expiratory volume in one second; FVC: forced vital capacity; LAMA: long-acting muscarinic antagonists; LABA: long-acting β2-agonists; ICS: inhaled corticosteroids.

## Data Availability

Data will be available upon request.
